# *Myocilin* Mutations Are Not a Major Cause of Primary Congenital Glaucoma in Iranian Patients

**Published:** 2010-04

**Authors:** Elahe Elahi, Mehrnaz Narooie-Nejhad, Fatemeh Suri, Shahin yazdani

**Affiliations:** 1 School of Biology, College of Science, University of Tehran, Tehran, Iran; 2 Department of Biotechnology, College of Science, University of Tehran, Tehran, Iran; 3 Center of Excellence in Biomathematics, Statistics and Computer Science, University of Tehran, Tehran, Iran; 4 National Institute of Genetic Engineering and Biotechnology, Tehran, Iran; 5 Ophthalmic Research Center, Shahid Beheshti University of Medical Sciences, Tehran, Iran

**Keywords:** Primary Congenital Glaucoma, *Myocilin*

## Abstract

**Purpose:**

To assess the frequency of mutations in the *Myocilin (MYOC)* gene in Iranian patients affected with primary congenital glaucoma (PCG).

**Methods:**

The individuals evaluated herein are among a larger cohort of 100 patients who had previously been screened for *CYP1B1* mutations. Eighty subjects carried mutations in *CYP1B1*, but the remaining 20 patients who did not, underwent screening for *MYOC* mutations for the purpose of the study. *MYOC* exons in the DNA were polymerase chain reaction (PCR) amplified and sequenced. Sequencing was performed using PCR primers, the ABI big dye chemistry and an ABI3730XL instrument. Sequences were analyzed by comparing them to reference *MYOC* sequences using the Sequencher software.

**Results:**

Four *MYOC* sequence variations were observed among the patients, but none of them were considered to be associated with disease status. Three of these variations were single nucleotide polymorphisms already reported not to be disease causing, the fourth variation created a synonymous codon and did not affect any amino acid change.

**Conclusion:**

In this cohort, *MYOC* mutations were not observed in any Iranian subject with PCG. It is possible that in a larger sample, a few subjects carrying disease causing *MYOC* mutations could have been observed. But our results show that the contribution of *MYOC* to PCG status in Iran is small if any.

## INTRODUCTION

Glaucoma affects more than 60 million people worldwide and is the second leading cause of blindness.^1^ It has traditionally been categorized based on etiology, anatomy of the anterior chamber angle, and age of onset.^2^ Two of the major forms of the disease are primary open angle glaucoma (POAG; OMIM 137760) and primary congenital glaucoma (PCG; OMIM 231300). PCG is a severe form of glaucoma that manifests at birth or early childhood and is associated with developmental defects in the trabecular meshwork.^3^ POAG is the most common form of glaucoma in Western countries and usually affects individuals past the age of 40 years.^2^ Genetic analysis has identified more than ten loci linked to POAG, attesting to its high genetic heterogeneity.^4^ Genes at three of these loci have been identified: *Myocilin* (*MYOC* at GLC1A; OMIM 601652), *Optineurin* (*OPTN* at GLC1E; OMIM 602432), and WD repeat containing protein 36 (*WDR36* at GLC1G; OMIM 609669).^5^–^7^ Mutations in *MYOC* have been observed more frequently than mutations in *OPTN* and *WDR36*. Three loci have been found for PCG, and genes at two of these loci have been identified: the *Cytochrome P4501B1* gene (*CYP1B1* at GLC3A; OMIM 601771) and the Latent Transforming Growth Factor (TGF) Beta Binding Protein 2 gene (*LTBP2* at GLC3C; OMIM 602091).^8^,^9^ *CYP1B1* mutations are most common, and have been observed in approximately 70% of Iranian PCG patients.^10^ Although *CYP1B1* is generally associated with PCG, several studies have reported *CYP1B1* mutations in POAG patients and it is now apparent that this gene can also cause POAG, particularly the juvenile onset form of the disease (juvenile open angle glaucoma, JOAG).^11^–^14^ Less often, *MYOC* mutations have been observed in PCG patients.^15^–^17^ *MYOC* mutations were sometimes observed in conjunction with *CYP1B1* mutations and sometimes without *CYP1B1* mutations. The biological significance of these observations is that some cases of PCG and POAG may share a common etiology.^18^ Herein, we report data on the frequency of *MYOC* mutation among Iranian PCG patients.

## METHODS

This study was performed in accordance with the Declaration of Helsinki and with approval by the Ethics Board of the National Institute of Genetic Engineering and Biotechnology in Iran, and the University of Tehran. Informed consent was obtained from all patients or their guardians. Originally, 123 unrelated Iranian PCG patients were recruited for genetic studies.^10^,^19^ Slitlamp biomicroscopy, intraocular pressure (IOP) measurement, gonioscopic evaluation of the angle, fundus examination, and perimetry had been performed whenever possible. PCG manifested by IOP ≥21 (range, 21–56) mmHg in at least one eye before treatment, corneal edema and/or opacification, Descemet’s membrane rupture, megalocornea (corneal diameter exceeding 12 mm), and optic nerve head changes suggestive of glaucoma including high cup/disc ratio or neural rim thinning or notching. The cup/disc ratio of affected eyes ranged from 0.3 to total (average value, 0.58). Mutation screening for *CYP1B1* was performed by direct sequencing and 80 patients among the initial 123 cases, were shown to have mutations in this gene.^10^,^19^ Twenty of the remaining 43 patients were available for further study. DNA was extracted from these 20 patients and the three *MYOC* exons were amplified by polymerase chain reaction (PCR) and subsequently screened for mutations by direct sequencing. Sequencing was performed using PCR primers and the ABI big dye chemistry and an ABI3730XL instrument (Applied Biosystems, Foster City, USA). Sequences were analyzed by comparing them to reference *MYOC* sequences (NT_004487, NM_000261, and NP_000252) using the Sequencher software (Gene Codes Corp., Ann Arbor, USA). PCR conditions and *MYOC* primer sequences are available upon request. For assessing the frequency of *MYOC* mutations, data on the 80 patients with *CYP1B1* mutations and the 20 patients without *CYP1B1* mutations in whom *MYOC* was sequenced were considered. The 23 patients without *CYP1B1* mutations who were not available for *MYOC* screening were not included in the analysis.

## RESULTS

Overall 20 patients including ten male and 10 female subjects, all with age of onset of PCG less than 3 years, were studied. Sequencing *MYOC* exons revealed four sequence variations. Three of these: c.-83G>A, c.227G>A and IVS2+35G>A, were observed in heterozygous state each in 6 distinct individuals and in homozygous state in 1, 1 and 6 individuals respectively. All of these are single nucleotide polymorphisms (SNPs) that have already been reported not to be disease causing.^20^–^22^ The same variations have previously been observed as high frequency polymorphisms in Iranian JOAG patients.^14^ The fourth variation, c.1041T>A, resulting in Y347Y, is also very unlikely to be associated with disease status because it creates a synonymous codon and does not cause any amino acid change; it was observed in the heterozygous state in one subject.

## DISCUSSION

We have ascertained that *MYOC* is not a major cause of PCG among Iranian patients. Although this gene was sequenced in only 20 patients and disease causing mutations were not found, these 20 patients are among 100 subjects whose status regarding *CYP1B1* is already known.^10^,^19^ Of the 100 patients, 80 have been shown to harbor disease causing *CYP1B1* mutations and are unlikely to simultaneously carry *MYOC* mutations. Therefore, it can reasonably be surmised that mutations in *MYOC* mutations were not the cause of PCG in 100 recruited patients. Studies comparable to ours have been reported on PCG patients from India and China. In studies from India, four among approximately 150 patients (*MYOC* screened in 72; the remaining carried *CYP1B1* mutations) in one study,^15^ and five among 200 subjects in another report^16^ carried *MYOC* mutations. In a study from China, three patients among 116 subjects harbored *MYOC* mutations.^17^ It is possible that in a larger cohort of Iranian PCG patients, a few individuals carrying disease causing *MYOC* mutations would have been identified. But clearly, the results of this study show that the contribution of *MYOC* to PCG status in Iran is small if any. Notably the contribution of *CYP1B1*, which is usually associated with PCG, to JOAG has been shown to be very significant in Iran.^14^,^23^ In fact, *MYOC* and *CYP1B1* may contribute equally to JOAG status among Iranian patients.^14^ The explanation for this may be that some cases of JOAG may be late onset forms of PCG rather than early onset forms of POAG. Fewer cases of PCG may share a common etiology with classic open angle glaucoma.

Since *MYOC* and *CYP1B1* mutations have been observed in the same individual in a few cases, a digenic mode of pathogenesis for glaucoma has sometimes been proposed.^15^,^18^ To the best of our knowledge, the *CYP1B1* mutation in such patients has always been R368H.^15^,^16^ The proposed digenic mode of pathogenesis for PCG should be considered with caution because it is not absolutely clear that the R368H mutation in *CYP1B1* actually is a disease associated variation. Its frequency in PCG affected and control groups in India has been reported to be similar.^11^ We have recently made the same observation among Iranian PCG patients and control individuals (unpublished data). We have also noted that the variation found in PCG patients harboring only one mutated *CYP1B1* allele is frequently R368H, suggesting that it may be a neutral polymorphism. Notably, both observations of a *MYOC* mutation coexisting with a R368H *CYP1B1* allele were made in patients from India.^15^,^16^ These observations emphasize that it is unclear whether *MYOC* and *CYP1B1* work in a common pathway toward PCG. The manner in which mutations in *MYOC* may affect PCG status remains unknown.
